# Development and validation of a risk-score model for opioid overdose using a national claims database

**DOI:** 10.1038/s41598-022-09095-y

**Published:** 2022-03-23

**Authors:** Kyu-Nam Heo, Ju-yeun Lee, Young-Mi Ah

**Affiliations:** 1 College of Pharmacy and Research Institute of Pharmaceutical Sciences, Seoul National University, Seoul, 08826 Republic of Korea; 2 College of Pharmacy, Yeungnam University, Gyeongsan, Gyeongbuk 38541 Republic of Korea

**Keywords:** Health care, Neurology

## Abstract

Opioid overdose can be serious adverse effects of opioid analgesics. Thus, several strategies to mitigate risk and reduce the harm of opioid overdose have been developed. However, despite a marked increase in opioid analgesic consumption in Korea, there have been no tools predicting the risk of opioid overdose in the Korean population. Using the national claims database of the Korean population, we identified patients who were incidentally prescribed non-injectable opioid analgesic (NIOA) at least once from 2017 to 2018 (N = 1,752,380). Among them, 866 cases of opioid overdose occurred, and per case, four controls were selected. Patients were randomly allocated to the development (80%) and validation (20%) cohort. Thirteen predictive variables were selected via logistic regression modelling, and a risk-score was assigned for each predictor. Our model showed good performance with c-statistics of 0.84 in the validation cohort. The developed risk score model is the first tool to identify high-risk patients for opioid overdose in Korea. It is expected to be applicable in the clinical setting and useful as a national level surveillance tool due to the easily calculable and identifiable predictors available from the claims database.

## Introduction

Opioids are considered crucial for pain relief; however, they are associated with significant harms such as overdose^1^. In the USA, along with the rise in opioid prescription since 1999, opioid-related overdose admissions had quadrupled by 2010^2^. During 1999‒2017, 702,568 drug-related deaths occurred, of which two-thirds were opioid-related^3^.

US regulatory authorities declared the opioid crisis a national public health emergency^4^. To encourage safe use of opioids, the Centers for Disease Control and Prevention published guidelines on safe opioid prescription practice in primary care settings^5,6^. Risk evaluation and mitigation strategies and prescription drug monitoring programs were implemented to encourage safe opioid use. Consequently, a decrease in opioid prescription was observed from 2012, and death by prescription opioids had decreased by 13.5% in 2017‒2018^7^. However, overdose deaths involving opioids are still on the rise, and the proportion of drug-related overdose deaths due to illicit opioids has risen sharply since 2017^8^. This phenomenon might be related to the unexpected interruption of opioid prescription due to the increased prescription burden of physicians. Thus, according to experts, prescription restriction policies should be applied with caution, and each patient’s situation should be considered^9,10^. For instance, patients inevitably in need of opioids, such as those with malignant pain, should receive opioid prescriptions. Therefore, to prevent opioid-related adverse outcomes, it is important to assess the individual patient risk rather than to regulate opioid prescriptions according to uniform standards.

In this context, several models for predicting opioid overdose have been reported. These included a risk index to identify patients with a risk of overdose or serious opioid-induced respiratory depression^11^ and a machine learning algorithm to predict opioid overdose^12^. However, most such studies were conducted in North America^11–17^. Considering that the healthcare system is key in prescription practice and healthcare utilisation^18,19^, and that ethnicity influences opioid-related negative outcomes^20^, these tools should be developed or validated in domestic populations.

Although Korea has had few issues regarding opioid overdose because opioid use is lower than in other countries, a significant increase in opioid prescription trends has been observed in recent years (347.5 prescriptions/1000 person in 2009 vs. 531.3 prescriptions/1000 person in 2019)^21^. Also, the prevalence of chronic use of opioid, a well-known risk factor for overdose, has sharply increased (strong opioids: 0.04% in 2002 to 0.24% in 2015)^22^. A multicentre study reported that 21% of patients on chronic opioid therapy for more than three months had instances of inappropriate or excessive opioid use^23^. To ameliorate the opioid-related problem due to opioid prescription increase, a program examining opioid prescriptions for physicians has recently been established in Korea^24^. However, there are no tools that can predict the risk of opioid overdose for an individual patient in the Korean population.

In this study, we aimed to develop and validate a predictive risk-score model for opioid overdose among Korean patients prescribed non-injectable opioid analgesic (NIOA). We used this model to stratify Korean patients prescribed NIOA into subgroups with similar risks for opioid overdose.

## Methods

### Study design and database

This study was a retrospective, nested case‒control study that used the National Health Insurance Claims Database of the Korean Health Insurance Review Service (HIRA) from 2016 to 2018. In Korea, it is compulsory for all citizens to enrol in the national health insurance system, and all medical claims are electronically submitted to the HIRA, which covers almost 98% of the entire population. Thus, these data are representative of the population^25^. The database contains detailed information on basic demographic characteristics, healthcare utilisation, medical procedures, prescription, and disease diagnosis, based on the International Statistical Classification of Diseases and Related Health Problems, Tenth Revision (ICD-10). This study was approved by the Yeungnam University Institutional Review Board (approval number: YU 2019-01-001). The informed consent from the participants was waived by the Yeungnam University Institutional Review Board because this study used anonymized data retrospectively, and there was no or minimal risk of harm to the participants. All methods were performed according to relevant guidelines and regulations.

### Study participants and outcome definition

First, adult patients who initiated NIOA use during 2017‒2018 (N = 1,752,380) were defined as the NIOA cohort, and the date of the first prescription was the cohort entry date. We excluded patients with NIOA prescriptions in 2016. We defined NIOA as oral or transdermal opioid analgesics (ATC code: N02A and R05DA04), excluding tramadol, and excluded injectable formulations.

Among them, the case group with opioid overdose events was defined as follows: (A) those with occurrence of an opioid-related poisoning event, (B) those with occurrence of emergency department (ED) visits involving a naloxone injection and active NIOA prescriptions within 180 days before the event (those with naloxone injection for the first developed stroke or within the context of a surgical procedure were not included). When a patient had more than one event, we gave a higher priority to outcome (A) than to (B). When outcome (A) occurred, we set the index date as the date of occurrence; otherwise, the date of occurrence of outcome (B) was set as the index date. Additionally, we classified the cases as severe when central respiratory depression was concurrently confirmed (Supplementary Table 1). We excluded patients with diagnosis codes for (A) or naloxone injection before the entry date to identify a new event. Patients who had naloxone injection after the entry date, but who were not classified as the case group, were also excluded.

In a ratio of 1:4, control groups were selected among patients without any aforementioned event, using the exact-match method, based on cohort-entry date and follow-up duration (duration between the date of initial opioid prescription and the date of the last medical record).

### Variables

The dependent variable for prediction was opioid overdose at the index date. Variables previously identified as risk factors or that were likely to be related to opioid overdose were selected as candidate predictors^14,17,26^.

The assessed variables were demographics (age, sex, and insurance status), baseline comorbidities (mental health disorders and other medical diagnoses), cause of pain at the initiation of NIOA, healthcare utilisation at baseline (hospitalisation period, number of ED visits), NIOA use pattern during the month prior to the index date (main ingredient, number of extended-release and long-acting [ER/LA] opioids, persistence of NIOA use, mean daily milligrams of morphine equivalents [MME]), other medications used during the month prior to the index date (anxiolytics, anticonvulsants, antidepressants, antipsychotics, benzodiazepines, gabapentinoids, muscle relaxants, non-opioid analgesics, naltrexone, other hypnotics, stimulants, and tramadol) (Supplementary Table 2), number of prescribers, and number of prescriptions for NIOA from cohort-entry date to index date. Individual mean daily MME was calculated as the sum of the total MMEs prescribed 1 month prior to the index date, divided by the number of days covered with NIOA prescriptions. Based on the continuity of the NIOA use 1 month before the index date, we classified the persistence of NIOA use as follows: (1) none: no active NIOA prescription for 2 months (− 60 to − 1 days), (2) new: new active NIOA prescription 1 month (− 30 to − 1 days), (3) past: active NIOA prescription ended before 1 month (− 60 to − 31 days), and (4) persistent: active NIOA prescription for 2 months (− 60 to − 1 days) prior to the event.

We assumed no missing values for the measured variables. Since we utilised a claim database, it was not possible to determine whether the absence of records, such as prescription or diagnosis records, was due to missing data. Therefore, we deemed the absence of record as the absence of a corresponding condition. We confirmed that there were no missing values for demographic factors, such as age, sex, and insurance status.

### Derivation of risk-score model

First, we randomly split the data into the development cohort (80%) and validation cohort (20%), stratified by outcome. To select the variables to be included in the prediction model, the following process was performed. The frequencies of candidate predictors were evaluated, and items with a prevalence of less than 1% were excluded. The excluded items were moderate to severe liver disease, sleep apnoea, stimulant use, and naltrexone use. We examined the multicollinearity between variables using the variance inflation factor. Next, variables with α ≤ 0.1 in the univariable logistic regression analysis were selected as potential predictors of opioid overdose. During this process, sex, myocardial infarction, rheumatic disease, and severe renal disease which did not met the criteria were excluded. To select more efficient variables, we utilised the Akaike Information Criterion (AIC) and performed stepwise selection through three repeated five-fold cross-validation steps^27^. Finally, when variables that were related to each other coexisted, we carefully modified our final list of variables, considering their applicability in the clinical field. For instance, benzodiazepine use was selected over anxiety disorder because use of benzodiazepine or possession of a medication could be easily identified by the interviewer, while the patient may be reluctant to reveal anxiety disorder to healthcare providers.

Multivariable logistic regression with the final list of variables was performed for the entire development cohort. Then, the risk-score model was developed through the risk-score assignment for each variable, by multiplying the β coefficient of each variable by 10 and rounding it to the nearest integer. Individual patient risk-scores were calculated using this model. We also created a simple prediction model that could be easily and accurately identified in a primary care setting. For this purpose, information that the patient may be reluctant to relay to the clinician or may not recall correctly, such as substance use disorder, number of NIOA prescriptions, and number of ED visits at baseline, were excluded from the full model.

Performance of the model was evaluated for discrimination and calibration in the validation cohort^28^. The c-statistic or area under the receiver operating characteristic curve was measured to quantify the discrimination performance. Calibration was evaluated qualitatively by constructing a calibration plot.

Considering the risk-score distribution of individuals and predicted probability in the development cohort, we classified participants into low-risk, intermediate-risk, and high-risk groups. The cut-off point for the intermediate-risk group was determined using the Youden index, which balances sensitivity and specificity^29^. The cut-off for the high-risk group was arbitrary determined by the score corresponding to the top 10% of all the scores obtained, as described previously^12^. Sensitivity, specificity, and positive- and negative-likelihood ratios were evaluated for these cut-off value. We also evaluated the distribution of severe cases of opioid overdose in each risk group.

We applied the developed prediction model with the strict outcome definition of limiting the active NIOA prescription window to 30 days before the index date, instead of 180 days, when identifying cases with outcome (B) and confirmed the performance in the total cohort.

### Sample size calculation

Sample size was calculated as described previously^30^. As we planned to match the case and control groups at a ratio of 1:4, the overall outcome proportion was set at 0.2. We targeted the mean absolute prediction error, R^2^Nagelkerke, and shrinkage factors as 0.05, 0.05, and 0.9, respectively. As we expected to develop a parsimonious model, the number of predictor parameters was set at 18. The event size required for model development was calculated to be 630 cases. Thus, the total of 690 cases in the development cohort provided adequate statistical power.

### Statistical analysis

For descriptive statistics, we used percentage or mean (standard deviation). The χ^2^ test or Fisher’s exact test was applied to compare categorical variables between groups, while *t* tests were used to compare continuous variables between groups. Logistic regression was performed to assess the association between the variables and outcomes. Statistical significance was determined at p-value less than 0.05. Statistical analyses were performed using SAS version 9.4 (SAS Institute, Cary, NC).

## Results

### Clinical characteristics of the development cohort

**Figure 1:**
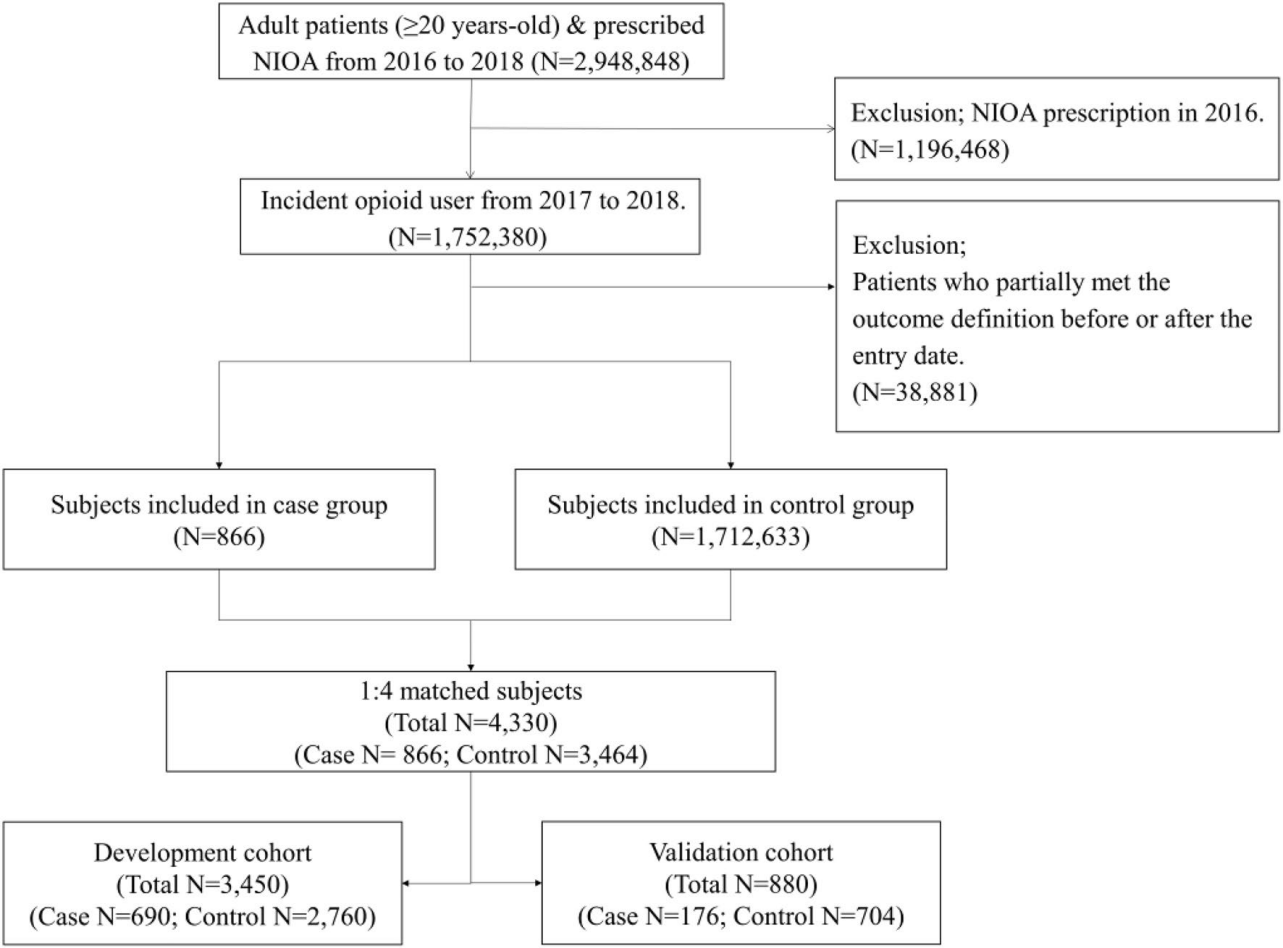
Patient-selection flow chart. *NIOA* non-injectable opioid analgesics.

From 1 January 2017 to 31 December 2018, we identified 866 case patients (0.05%) and 3464 matched control patients among the 1,752,380 patients initiated NIOA (Fig. 1). Patients in the development and validation cohorts had similar characteristics and outcome distributions (Supplementary Table 3). The mean ± standard deviation of age (development cohort: 60.5 ± 17.8 vs. validation cohort: 61.0 ± 18.3), Charlson comorbidity index (CCI) score (3.1 ± 2.7 vs. 3.0 ± 2.7), proportion of male patients (46.4% vs. 44.7%), beneficiaries with medical aid or national meritorious service (NMS) (10.8% vs. 12.0%), and patients with comorbidities, such as cancer (21.2% vs. 21.3%) and substance use disorder (1.3% vs. 1.4%), were not statistically significantly different between the development and validation cohorts.

In the development cohort, compared to the control group, the patients in the case group were older (69.3 ± 14.6 vs. 58.2 ± 17.8, p < 0.001) and had higher CCI scores (4.4 ± 2.8 vs. 2.7 ± 2.6, p < 0.001), and the proportion of medical aid or NMS beneficiaries was higher (21.4% vs. 8.2%, p < 0.001) (Table 1). Patients in the case group were more likely to be diagnosed with mood disorders, anxiety, schizophrenia, substance use disorder, cerebrovascular disease, dementia, pancreatitis, and respiratory disease. The proportion of patients prescribed each of the concurrent medications, such as benzodiazepine, was also higher in the case group (Table 2).

**Table 1:** Baseline characteristics of study participants according to outcomes in the development and validation cohorts.

Variables, N (%)	Development cohort			Validation cohort		
	Control (N = 2760)	Case (N = 690)	p-value	Control (N = 704)	Case (N = 176)	p-value
**Age group, mean ± SD**	58.2 ± 17.8	69.3 ± 14.6	< 0.001	58.7 ± 18.5	69.9 ± 14.1	< 0.001
20–50 years	864 (31.3)	65 (9.4)	< 0.001	210 (29.8)	16 (9.1)	
50–75 years	1302 (47.2)	312 (45.2)		334 (47.4)	75 (42.6)	
≥ 75 years	594 (21.5)	313 (45.4)		160 (22.7)	85 (48.3)	
**Male**	1276 (46.2)	325 (47.1)	0.682	306 (43.5)	87 (49.4)	0.154
**CCI score, mean ± SD**	2.7 ± 2.6	4.4 ± 2.8	< 0.001	2.7 ± 2.6	4.6 ± 2.8	< 0.001
0–2	1605 (58.2)	180 (26.1)	< 0.001	413 (58.7)	43 (24.4)	< 0.001
3–4	591 (21.4)	207 (30.0)		151 (21.4)	54 (30.7)	
5–6	307 (11.1)	155 (22.5)		80 (11.4)	37 (21)	
≥ 7	257 (9.3)	148 (21.4)		60 (8.5)	42 (23.9)	
**Health insurance type**						
Medical insurance	2535 (91.8)	542 (78.6)	< 0.001	637 (90.5)	137 (77.8)	< 0.001
Medical aid or NMS	225 (8.2)	148 (21.4)		67 (9.5)	39 (22.2)	
**Cancer**						
No	2228 (80.7)	491 (71.2)	< 0.001	578 (82.1)	115 (65.3)	< 0.001
Non-metastatic cancer	380 (13.8)	133 (19.3)		91 (12.9)	40 (22.7)	
Metastatic cancer	152 (5.5)	66 (9.6)		35 (5.0)	21 (11.9)	
**Comorbid disease**						
Mood disorder	444 (16.1)	253 (36.7)	< 0.001	122 (17.3)	65 (36.9)	< 0.001
Anxiety	530 (19.2)	272 (39.4)	< 0.001	134 (19.0)	58 (33.0)	< 0.001
Schizophrenia	28 (1.0)	29 (4.2)	< 0.001	11 (1.6)	7 (4.0)	0.067^a^
Substance use disorder	17 (0.6)	27 (3.9)	< 0.001	4 (0.6)	8 (4.5)	< 0.001^a^
Myocardial infarction	51 (1.8)	17 (2.5)	0.298	16 (2.3)	7 (4.0)	0.195^a^
Heart failure	193 (7.0)	108 (15.7)	< 0.001	60 (8.5)	32 (18.2)	< 0.001
Diabetes mellitus	786 (28.5)	327 (47.4)	< 0.001	194 (27.6)	79 (44.9)	< 0.001
Hypertension	1165 (42.2)	475 (68.8)	< 0.001	318 (45.2)	114 (64.8)	< 0.001
PVD	453 (16.4)	193 (28.0)	< 0.001	116 (16.5)	51 (29.0)	< 0.001
CVD	315 (11.4)	265 (38.4)	< 0.001	93 (13.2)	61 (34.7)	< 0.001
Dementia	240 (8.7)	183 (26.5)	< 0.001	60 (8.5)	43 (24.4)	< 0.001
Pancreatitis	147 (5.3)	56 (8.1)	0.005	40 (5.7)	16 (9.1)	0.098
Respiratory disease	1262 (45.7)	368 (53.3)	< 0.001	328 (46.6)	90 (51.1)	0.280
Rheumatic disease	165 (6.0)	57 (8.3)	0.029	50 (7.1)	14 (8.0)	0.697
Peptic ulcer disease	880 (31.9)	279 (40.4)	< 0.001	203 (28.8)	66 (37.5)	0.026
Liver disease (moderate to severe)	24 (0.9)	10 (1.4)	0.168	7 (1.0)	2 (1.1)	1^a^
Renal disease (severe)	53 (1.9)	16 (2.3)	0.504	10 (1.4)	3 (1.7)	0.731^a^
Sleep apnoea	10 (0.4)	1 (0.1)	0.365	1 (0.1)	0 (0)	1^a^
**No. of emergency department visit at baseline**						
0	2167 (78.5)	411 (59.6)	< 0.001	555 (78.8)	98 (55.7)	< 0.001
1–3	561 (20.3)	244 (35.4)		144 (20.5)	68 (38.6)	
≥ 4	32 (1.2)	35 (5.1)		5 (0.7)	10 (5.7)	
**Cumulative duration of admission at baseline**						
0	1814 (65.7)	316 (45.8)	< 0.001	449 (63.8)	75 (42.6)	< 0.001
1–7	430 (15.6)	112 (16.2)		115 (16.3)	30 (17.0)	
≥ 8	516 (18.7)	262 (38.0)		140 (19.9)	71 (40.3)	

*SD* standard deviation, *CCI* Charlson comorbidity index, *NMS* National Meritorious Service, *PVD* peripheral vascular disease, *CVD* cerebrovascular disease.

^a^Fisher’s exact test.

**Table 2:** Medication use pattern of study participants according to outcomes in the development and validation cohorts.

Variables, N (%)	Development cohort			Validation cohort		
	Control (N = 2760)	Case (N = 690)	p-value	Control (N = 704)	Case (N = 176)	p-value
**Concurrent medication**						
Anxiolytics	64 (2.3)	35 (5.1)	< 0.001	21 (3.0)	6 (3.4)	0.769
Anticonvulsant	57 (2.1)	85 (12.3)	< 0.001	13 (1.8)	27 (15.3)	< 0.001
Antidepressants	286 (10.4)	179 (25.9)	< 0.001	78 (11.1)	51 (29.0)	< 0.001
Antipsychotics	109 (3.9)	100 (14.5)	< 0.001	35 (5.0)	29 (16.5)	< 0.001
Benzodiazepines	390 (14.1)	217 (31.4)	< 0.001	105 (14.9)	54 (30.7)	< 0.001
Gabapentinoids	207 (7.5)	140 (20.3)	< 0.001	47 (6.7)	37 (21.0)	< 0.001
Muscle relaxant	493 (17.9)	172 (24.9)	< 0.001	115 (16.3)	43 (24.4)	0.012
Non-opioid analgesics	1681 (60.9)	501 (72.6)	< 0.001	408 (58.0)	135 (76.7)	< 0.001
Naltrexone	2 (0.1)	0 (0)	0.479	0 (0)	0 (0)	–
Other hypnotics	156 (5.7)	100 (14.5)	< 0.001	34 (4.8)	27 (15.3)	< 0.001
Stimulants	0 (0)	3 (0.4)	< 0.001	1 (0.1)	2 (1.1)	0.104^a^
Tramadol	575 (20.8)	250 (36.2)	< 0.001	131 (18.6)	74 (42.0)	< 0.001
**NIOA use pattern**						
**Cause of initiations**						
Traumatic injury	389 (14.1)	112 (16.2)	0.002	88 (12.5)	25 (14.2)	0.663
Surgery	655 (23.7)	122 (17.7)		172 (24.4)	38 (21.6)	
Other	1716 (62.2)	456 (66.1)		444 (63.1)	113 (64.2)	
**Ingredients**						
Buprenorphine	269 (9.7)	126 (18.3)	< 0.001	51 (7.2)	27 (15.3)	< 0.001
Codeine	518 (18.8)	112 (16.2)	0.123	123 (17.5)	21 (11.9)	< 0.001
Dihydrocodeine	22 (0.8)	7 (1.0)	0.576	3 (0.4)	1 (0.6)	1^a^
Fentanyl	242 (8.8)	124 (18.0)	< 0.001	51 (7.2)	35 (19.9)	< 0.001
Hydrocodone	22 (0.8)	3 (0.4)	0.316	8 (1.1)	3 (1.7)	0.467^a^
Hydromorphone	49 (1.8)	18 (2.6)	0.156	8 (1.1)	3 (1.7)	0.467^a^
Morphine	9 (0.3)	7 (1.0)	0.017	1 (0.1)	2 (1.1)	0.104^a^
Oxycodone	222 (8.0)	137 (19.9)	< 0.001	61 (8.7)	44 (25.0)	< 0.001
Tapentadol	24 (0.9)	8 (1.2)	0.477	1 (0.1)	4 (2.3)	0.007
**No. of ER/LA opioid**						
0	2113 (76.6)	374 (54.2)	< 0.001	570 (81.0)	86 (48.9)	< 0.001
1	572 (20.7)	263 (38.1)		118 (16.8)	81 (46)	
≥ 2	75 (2.7)	53 (7.7)		16 (2.3)	9 (5.1)	
**Persistence of use before index date**						
No	1251 (45.3)	197 (28.6)	< 0.001	361 (51.3)	50 (28.4)	< 0.001
Past	321 (11.6)	67 (9.7)		81 (11.5)	17 (9.7)	
New	784 (28.4)	219 (31.7)		186 (26.4)	64 (36.4)	
Persistent	404 (14.6)	207 (30.0)		76 (10.8)	45 (25.6)	
**No. of prescriber**						
1	2507 (90.8)	533 (77.2)	< 0.001	646 (91.8)	119 (67.6)	< 0.001
2	210 (7.6)	111 (16.1)		54 (7.7)	47 (26.7)	
≥ 3	43 (1.6)	46 (6.7)		4 (0.6)	10 (5.7)	
**No. of prescription**						
1–2	2345 (85)	455 (65.9)	< 0.001	584 (83.0)	100 (56.8)	< 0.001^a^
3–6	320 (11.6)	133 (19.3)		100 (14.2)	55 (31.3)	
7–9	57 (2.1)	41 (5.9)		13 (1.8)	9 (5.1)	
≥ 10	38 (1.4)	61 (8.8)		7 (1.0)	12 (6.8)	
**Daily MME**						
0–19	2380 (86.2)	478 (69.3)	< 0.001	625 (88.8)	114 (64.8)	< 0.001
20–49	216 (7.8)	114 (16.5)		46 (6.5)	33 (18.8)	
≥ 50	164 (5.9)	98 (14.2)		33 (4.7)	29 (16.5)	

*NIOA* non-injectable opioid analgesics, *ER/LA* extended-release and long-acting, *MME* morphine milligram equivalent.

^a^Fisher’s exact test.

Initiation of NIOA due to surgery was more frequent in the control group (23.7%) than those in the case group (17.7%). NIOA use patterns, such as prescription frequency, number of prescribers, and proportion of the patients prescribed ER/LA NIOA were significantly different between the groups. Compared to the control group, the patients in the case group were more frequently prescribed ER/LA opioids (≥ 1 agent; 23.4% vs. 45.8%), prescribed a higher daily dose (≥ 20 MMEs; 13.8% vs. 30.7%), had more prescribers (≥ 2 prescribers; 9.2% vs. 22.8%), and had a higher prescription frequency (≥ 3 times; 15.0% vs. 34.1%).

### Development of risk-score model for opioid overdose

The results of univariable and multivariable logistic regression analyses that were used to develop the risk screening scores for opioid overdose are summarised in Table 3 (results of univariable analysis of all candidate predictors are listed in Supplementary Table 4). Thirteen predictors were associated with the development of opioid overdose: age group, insurance status, cause of opioid prescription, three comorbidities (cerebrovascular disease, dementia, and substance use disorder), four classes of medication use (anticonvulsants, benzodiazepine, gabapentinoids, and tramadol), three types of NIOA use patterns (cause of initiations, ER/LA opioid use, and number of opioid prescriptions), and the number of ED visits at baseline. Among those variables, older age (≥ 75 years) (adjusted odds ratio [aOR] 3.47; 95% confidence interval [CI] 2.48–4.86), history of substance use disorder (aOR 4.67; 95% CI 2.34–9.34), exposure to anticonvulsants (aOR 5.00; 95% CI 3.34–7.47), and frequent opioid prescriptions (≥ 10) (aOR 6.07; 95% CI 3.79–9.72) were identified as factors markedly increasing the risk of opioid overdose. On the other hand, NIOA initiation due to surgical pain appeared to be a protective factor (aOR 0.62; 95% CI 0.49–0.80). Using the above-mentioned 13 variables, the screening score was developed, with a possible range of − 5 to 129.

**Table 3:** Development of the predictive risk-score model for opioid overdose.

Risk factor	Univariable OR (95% CI)	Multivariable aOR (95% CI)	β Coefficient	Score
**Age group**				
20–50	Reference	Reference	–	0
50–75	3.19 (2.41–4.22)	2.00 (1.46–2.73)	0.69	7
≥ 75	7.00 (5.26–9.33)	3.47 (2.48–4.86)	1.24	12
**Health insurance type**				
Medical insurance	Reference	Reference	–	0
Medical aid or NMS	3.08 (2.45–3.86)	1.55 (1.18–2.04)	0.44	4
**Comorbidities**				
CVD	4.84 (3.99–5.87)	2.5 (1.98–3.15)	0.91	9
Dementia	3.79 (3.06–4.70)	1.34 (1.03–1.75)	0.29	3
Substance use disorder	6.57 (3.56–12.13)	4.67 (2.34–9.34)	1.54	15
**No. of emergency department visit at baseline**				
0	Reference	Reference	–	0
1–3	2.29 (1.91–2.76)	1.53 (1.24–1.89)	0.42	4
≥ 4	5.77 (3.53–9.42)	2.51 (1.38–4.58)	0.92	9
**Concurrent medication**				
Anticonvulsants	6.66 (4.71–9.43)	5.00 (3.34–7.47)	1.61	16
Benzodiazepine	2.79 (2.30–3.38)	1.46 (1.16–1.83)	0.38	4
Gabapentinoids	3.14 (2.49–3.96)	1.64 (1.25–2.16)	0.49	5
Tramadol	2.16 (1.80–2.59)	1.34 (1.08–1.66)	0.29	3
**Cause of analgesics**				
Other^a^	Reference	Reference	–	0
Surgery	0.69 (0.56–0.86)	0.62 (0.49–0.80)	− 0.47	− 5
**No. of ER/LA opioid**				
0	Reference	Reference	–	0
≥ 1^b^	2.76 (2.32–3.28)	1.57 (1.27–1.94)	0.45	5
**No. of opioid prescription**				
1–2	Reference	Reference	–	0
3–6	2.14 (1.71–2.69)	1.68 (1.3–2.18)	0.52	5
7–9	3.71 (2.45–5.61)	2.36 (1.48–3.76)	0.86	9
≥ 10	8.27 (5.45–12.56)	6.07 (3.79–9.72)	1.80	18

*CI* confidence interval, *NMS* National Meritorious Service, *CVD* cerebrovascular disease, *ER/LA* extended-release and long-acting.

^a^Traumatic injury was collapsed into other category.

^b^For multivariable logistic regression model, ≥ 2 ER/LA opioid was collapsed into ≥ 1 ER/LA opioid.

**Figure 2:**
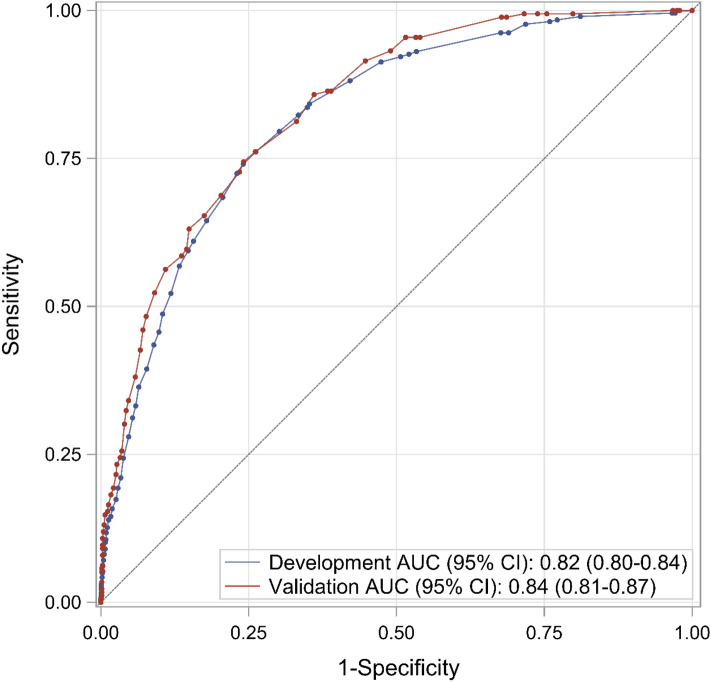
Receiver operating characteristic curve of predictive risk-score model for opioid overdose. *AUC* area under the receiver operating characteristic curve, *CI* confidence interval.

**Figure 3:**
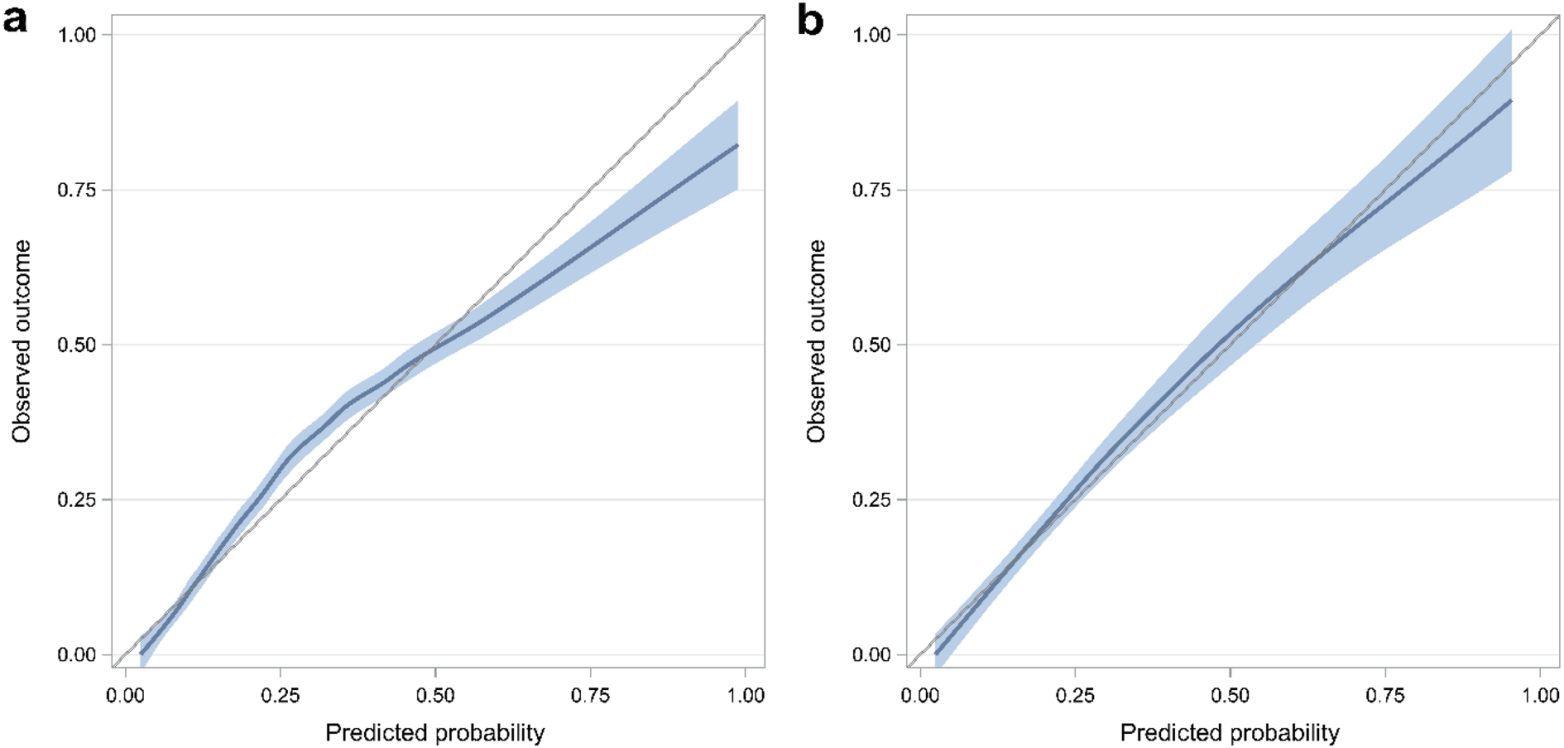
Calibration plot for predictive risk-score model for opioid overdose. (**a**) Calibration plot in the development cohort. (**b**) Calibration plot in the validation cohort.

The c-statistic of the screening score in development cohort was 0.82 (95% CI 0.80–0.84), while in the validation cohort, it was 0.84 (95% CI 0.81–0.87) (Fig. 2). The calibration plot indicated that the model fit the observed data well, as the prediction line was close to the perfect agreement line (Fig. 3).

When the full model was adjusted to a simple model after excluding variables that were difficult to identify in the primary care setting, including substance use disorder, number of NIOA prescriptions, and number of ED visits at baseline, the simple model showed good performance, although the performance on the ROC analysis was slightly reduced (c-statistic 0.81, 95% CI 0.77–0.84; ROC comparison, p < 0.001) (see Supplementary Figs. 1, 2, and Supplementary Table 5).

### Risk stratification

Table 4 shows the results of stratification into low- (< 18 points), intermediate- (18–31 points), and high-risk (≥ 32 points) groups in the development and validation cohorts. In the validation cohort, 579 (65.8%), 214 (24.3%), and 87 (9.9%) patients were classified as low, intermediate, and high risk, respectively, of which 45 (7.8%), 74 (34.6%), and 57 (65.5%) eventually developed opioid overdose, respectively. In each risk group, there were 14 (2.4%), 22 (10.3%), and 21 (24.1%) severe cases. When using the intermediate-risk threshold, the sensitivity and specificity of the model for predicting opioid overdose was 74.4% (95% CI 67.3‒80.7%) and 75.9% (95% CI 72.5‒79.0%), respectively, and the positive- and negative-likelihood ratios were 3.08 (95% CI 2.63–3.61) and 0.34 (95% CI 0.26–0.44), respectively. When using the high-risk threshold, the sensitivity and specificity were 32.4% (95% CI 25.5‒39.8%) and 95.7% (95% CI 94.0‒97.1%), respectively, while the positive- and negative-likelihood ratios were 7.60 (95% CI 5.04–11.45) and 0.71 (95% CI 0.64–0.78), respectively. The odds ratios of the intermediate-risk group and high-risk group, as compared to the low-risk group, were 6.27 (95% CI 4.14–9.50) and 22.55 (95% CI 13.18–38.56), respectively. The results of the simple model are presented in Supplementary Table 6.

**Table 4:** Prediction performance stratified by risk.

Performance metrics (full model)	Development cohort			Validation cohort		
	Low risk	Intermediate risk	High risk	Low risk	Intermediate risk	High risk
Total, N (%)	2274 (65.9)	813 (23.6)	363 (10.5)	579 (65.8)	214 (24.3)	87 (9.9)
Predicted score (range)	(− 5 to 17)	(18 to 31)	(32 to 129)	(− 5 to 17)	(18 to 31)	(32 to 129)
Actual overdose episodes, N (% of each subgroup)	179 (7.9)	296 (36.4)	215 (59.2)	45 (7.8)	74 (34.6)	57 (65.5)
Actual non-overdose episodes, N (% of each subgroup)	2095 (92.1)	517 (63.6)	148 (40.8)	534 (92.2)	140 (65.4)	30 (34.5)
Actual severe overdose episodes, N (% of each subgroup)	48 (2.1)	95 (11.7)	80 (22.0)	14 (2.4)	22 (10.3)	21 (24.1)
Actual non-severe overdose episodes, N (% of each subgroup)	2226 (97.9)	718 (88.3)	283 (78.0)	565 (97.6)	192 (89.7)	66 (75.9)
Sensitivity, % (95% CI)	–	74.1 (70.6–77.3)	31.2 (27.7–34.8)	–	74.4 (67.3–80.7)	32.4 (25.5–39.8)
Specificity, % (95% CI)	–	75.9 (74.3–77.5)	94.6 (93.7–95.5)	–	75.9 (72.5–79.0)	95.7 (94.0–97.1)
LR (+) (95% CI)	–	3.07 (2.84–3.33)	5.81 (4.80–7.04)	–	3.08 (2.63–3.61)	7.60 (5.04–11.45)
LR (−) (95% CI)	–	0.34 (0.30–0.39)	0.73 (0.69–0.77)	–	0.34 (0.26–0.44)	0.71 (0.64–0.78)
OR (95% CI)	Reference	6.70 (5.44–8.26)	17.00 (13.12–22.03)	Reference	6.27 (4.14–9.50)	22.55 (13.18–38.56)
% of all overdose episodes captured^a^	25.9%	42.9%	31.2%	25.6%	42.0%	32.4%
% of all severe overdose episodes captured ^b^	21.5%	42.6%	35.9%	24.6%	38.6%	36.8%

*CI* confidence interval, *LR* likelihood ratio, *OR* odds ratio.

^a^Development cohort: n = 690; validation cohort: n = 176.

^b^Development cohort: n = 223; validation cohort: n = 57.

The application results of the full model or simple model with strict outcome definitions showed similar trends (Supplementary Figs. 3–6, and Supplementary Tables 7, 8).

## Discussion

Using a national claim database comprising virtually the entire Korean adult population who had started NIOA, we developed and internally validated a risk-score model as a screening tool that can classify patients at high risk of opioid overdose. The risk screening tool comprised 13 items, including several well-documented risk factors related to opioid overdose^26,31–34^. We found that older age; beneficiaries with medical aid or NMS medical care; the existence of baseline comorbidities, such as substance use disorder, cerebrovascular disease, and dementia; exposure to medication with sedative properties; having a high number of NIOA prescriptions; use of ER/LA NIOA; and an ED visit before NIOA initiation predicted an increased risk of opioid overdose. On the other hand, use of NIOA for pain due to surgery showed a protective effect. Compared to the previous studies in the USA, which reported the incidence of opioid overdose as 0.4–0.6%^12,35^, the observed incidence of opioid overdose events in the population that initiated NIOA in our study was very low (0.05%).

Predictors showing the strongest association were the number of NIOA prescriptions within the period from the cohort entry date to the index date, with ≥ 10 NIOA prescriptions resulting in more than six times the risk (aOR 6.07; 95% CI 3.79‒9.72). While several previous studies found the number of opioid prescribers or pharmacies as risk factors for opioid overdose^13,26,36^, the number of opioid prescriptions, instead of the number of prescribers, was a predictor of opioid overdose in the present study. These results may be attributable to the health care system of Korea. Similar to previous studies, medical aid beneficiaries^32^, a prior history of substance use disorder^14,16,17,26,31,34^, ER/LA opioid prescription^14,34^, and exposure to medications acting on the central nervous system, such as benzodiazepines and gabapentinoids, increased the odds of opioid overdose^34,37–39^.

Since tramadol is not regulated as a controlled substance in Korea, we did not consider tramadol as a NIOA. Thus, patients prescribed only tramadol were not included in the study. However, among the participants included, tramadol use was evaluated as an additional medication in this study. While previous studies have identified tramadol as a protective factor against serious opioid-related overdoses^34^, we found that exposure to tramadol was associated with an increased risk of opioid overdose. However, this result should be cautiously interpreted.

The impact of age on opioid overdose is controversial. Hasegawa et al. showed that the risk of opioid overdose decreased in the elderly and increased in the middle-aged group compared to younger adults^32^, Bonhert et al. also found decreased risk with older age^40^, while Cho et al., Rose et al., and Khanna et al. reported increased risk with older age^15,39,41^. Our results showed that the odds for opioid overdose increased with age. Older individuals are more sensitive to opioids due to pharmacokinetic factors, such as reduced renal and hepatic clearance, which induce prolonged effects of opioids, followed by respiratory depression^42^.

Previously developed models for prediction of opioid overdose have reported c-statistics of between 0.69 and 0.91^12–17^. Our final model showed consistent, good discrimination and calibration with c-statistics of 0.84 (95% CI 0.81–0.87) in the validation cohort. While previous studies have developed prediction models with databases from specific populations (e.g., a veterans’ health administration database)^12,16^, we utilised a national claims database that included nearly 100% of the Korean population. This implies generalizability of our findings to all patients prescribed NIOA in Korea. The high prediction performance obtained in the validation cohort provided evidence of the strong internal validity of the study. However, this tool needs to be used with caution, considering the low prevalence of opioid overdose, as a low prevalence results in a low positive-predictive value and a high-negative predictive value. Taking this into consideration, this tool should not be used as a standalone tool to make decisions about initiation or modification of NIOA treatment, but should be used to identify patients requiring more intensive monitoring with respect to opioid overdose. By focusing preferentially on patients in the intermediate-to high-risk group, almost 75% cases of opioid overdose can be captured, saving time and resources for evaluating patients unlikely to develop serious adverse effects.

Our prediction model is expected to be applicable in the clinical setting and in national-level surveillance, because of its readily identifiable and easily calculable nature. In particular, a simple model that utilises variables that can be identified through interviews and prescription details is expected to be useful in primary care. We identified several modifiable predictors, such as NIOA prescription patterns and non-opioid medication use patterns that can predict future opioid overdose. In this regard, this tool can help clinicians identify patients at-risk of opioid overdose, and to assist in decision-making about reducing the risk of preventable adverse effects when evaluating the risks and benefits of the treatment.

Our study had several limitations. Due to the nature of the claims database, we could not investigate some important sociodemographic factors, such as family history, smoking behaviour, and alcohol consumption. Second, an individual’s actual NIOA intake could not be evaluated because only prescription records were available. Third, although we identified all patients eligible for our definition of opioid overdose, only a portion of patients without opioid overdose were included as control patients, because four control patients were assigned to each case. As a result, it was not possible to calculate positive- and negative-predictive values directly, which are subject to the prevalence of the outcome^43^. We also could not estimate the absolute incidence of opioid overdose in each risk group from our model. Finally, although our novel screening score had been internally validated, it should be externally validated in different data sources or in specific subgroups of patients to ensure generalizability in different settings^44^.

In conclusion, we have newly developed and internally validated a model for predicting opioid overdose in opioid incident users in Korea, based on the national claim database. This tool can stratify patients according to their risk of opioid overdose, allowing healthcare providers to focus medical resources on a limited number of patients. Ideally, this tool can also be utilised as a national-level surveillance tool to identify patients at risk of opioid overdose. To ensure the clinical usefulness of the model, external validation and prospective evaluation of the model in a real-world setting are needed.

## Supplementary Information

Supplementary Information.

## Data Availability

We used the national health insurance database in Korea, which was provided by Health Insurance Review and Assessment Service (HIRA) in South Korea (No. M20200325410). The databases are prohibited to transfer, rent or sale to any third party as well as the researcher who have been officially approved for database use. (Official website: https://opendata.hira.or.kr/home.do) Other researchers can request access to the data from the HIRA via the official website.
