# Supplementary material for: Development and validation of a risk-score model for opioid overdose using a national claims database
Source: Sci Rep. 2022 Mar 23;12:4974. doi: 10.1038/s41598-022-09095-y (PMC8943129; doi:10.1038/s41598-022-09095-y)
Supplement: Supplementary file 1 — Supplementary Information. [file 41598_2022_9095_MOESM1_ESM.docx]

**Supplementary Material**

Supplementary table 1. Diagnosis codes and condition to identify opioid overdose

| **Opioid overdose** | | |
| --- | --- | --- |
| **Definition** |  | **ICD-10 code** |
| Opioid-related poisoning event |  | T40.1 Poisoning by opium  T40.2 Poisoning by heroin  T40.3 Poisoning by other opioids  T40.4 Poisoning by methadone  T40.6 Poisoning by other and unspecified narcotics |
| **Severe opioid overdose** | | |
| **Definition** |  | **Condition** |
| Opioid overdose with confirmed central respiratory depression  (Any of described condition) |  | Claim with ICU admission |
|  |  | Claim with Mechanical ventilation |
|  |  | Claim with respiratory depression disease code  ICD-10 code: E03.5, J80, J96, R06.8, R09.0, R09.2, R40 |

ICD-10, international classification of diseases 10^th^ revision; ICU, intensive care unit

Supplementary table 2. ATC codes of drug of interest (Non-injectable)

| **Anxiolytics** | Buspirone (N05BE01), hydroxyzine (N05BB01)  ATC code not available: tandospirone (N05B) |
| --- | --- |
| **Anticonvulsants** | Phenobarbital (N03AA02), primidone (N03AA03), phenytoin (N03AB02), ethosuximide (N03AD01), carbamazepine (N03AF01), oxcarbazepine (N03AF02), rufinamide(N03AF03), valproic acid (N03AG01), vigabatrin(N03AG04), lamotrigine (N03AX09), topiramate (N03AX11), levetiracetam (N03AX14), zonisamide (N03AX15), stripentol (N03AX17), lacosamide (N03AX18), perampanel (N03AX22), |
| **Antidepressants** | Imipramine (N06AA02), clomipramine (N06AA04), amitriptyline (N06AA09), nortriptyline (N06AA10), doxepin (N06AA12), dothiepin (N06AA16), amoxapine (N06AA17), amineptine (N06AA19), maprotiline (N06AA21), quinupramine (N06AA23), fluoxetine (N06AB03), citalopram (N06AB04), paroxetine (N06AB05), sertraline (N06AB06), fluvoxamine (N06AB08), escitalopram (N06AB10), moclobemide (N06AG02), toloxatone (N06AG03), mianserine (N06AX03), trazodone (N06AX05), nefazodone (N06AX06), mirtazapine (N06AX11), bupropion (N06AX12), medifoxamine (N06AX13), tianeptine (N06AX14), venlafaxine (N06AX16), milnacipran (N06AX17), duloxetine (N06AX21), desvenlafaxine (N06AX23), vortioxetine (N06AX26) |
| **Antipsychotics** | Chlorpromazine (N05AA01), levomepromazine (N05AA02), perphenazine (N05AB03), prochlorperazine (N05AB04), trifluoperazine (N05AB06), thioridazine (N05AC02), mesoridazine (N05AC03), haloperidol (N05AD01), bromperidol (N05AD06), molindone (N05AE02), ziprasidone (N05AE04), flupentixol (N05AF01), chlorprothixene (N05AF03), thiothixene (N05AF04), zuclopenthixol (N05AF05), pimozide (N05AG02), loxapine (N05AH01), clozapine (N05AH02), olanzapine (N05AH03), quetiapine (N05AH04), nemonapride (N05AL), sulpiride (N05AL01), tiapride (N05AL03), amisulpride (N05AL05), lithium (N05AN01), risperidone (N05AX08), zotepine (N05AX11), aripiprazole (N05AX12), paliperidone (N05AX13)  ATC code not available: blonanserin (N05AX), nemonapride (N05AL) |
| **Benzodiazepines** | Clonazepam (N03AE01), diazepam (N05BA01), chlordiazepoxide (N05BA02), oxazepam (N05BA04), clorazepate (N05BA05), lorazepam (N05BA06), bromazepam (N05BA08), clobazam (N05BA09), alprazolam (N05BA12), pinazepam (N05BA14), fludiazepam (N05BA17), ethyl loflazepate (N05BA18), etizolam (N05BA19), clotiazepam (N05BA21), tofisopam (N05BA23), mexazolam (N05BA25), flurazepam (N05CD01), flunitrazepam (N05CD03), estazolam (N05CD04), triazolam (N05CD05), temazepam (N05CD07), midazolam (N05CD08), brotizolam (N05CD09), |
| **Gabapentinoids** | Gabapentin (N03AX12), pregabalin (N03AX16) |
| **Muscle relaxant** | Carisoprodol (M03BA02), methocarbamol (M03BA03), chlorzoxazone (M03BB03), orphenadrine (M03BC01), chlorphenesin (M03BX), thiocolchicoside (M03BX), baclofen (M03BX01), tizanidine (M03BX02), pridinol (M03BX03), tolperisone (M03BX04), thiocolchicoside (M03BX05), cyclobenzaprine (M03BX08), eperisone (M03BX09), dantrolene (M03CA01)  ATC code not available: afloqualone (M03BX), chlorphenesin (M03BX), |
| **Non-opioid analgesics** | ATC 4th levels: butylpyrazolidines (M01AA), acetic acid derivatives and related substances (M01AB), oxicams (M01AC), propionic acid derivatives (M01AE), fenamates (M01AG), coxibs (M01AH),  nabumetone (M01AX01), nimesulide (M01AX17), morniflumate (M01AX22),  ATC 3th levels: other analgesics and antipyretics (N02B), |
| **Naltrexone** | N07BB04 |
| **Other hypnotics** | Chloral hydrate (N05CC01), dichloralphenazone (N05CC04), zolpidem (N05CF02), eszopiclone (N05CF04) |
| **Stimulants** | Methylphenidate (N06BA04), modafinil (N06BA07), atomoxetine (N06BA09), armodafinil (N06BA13) |
| **Tramadol** | N02AX02, N02AJ13 |

ATC code, anatomical therapeutic chemical code

Supplementary table 3. Characteristics of the study participants in the development and validation cohorts

| **Variables, N (%)** |  | Development cohort (N=3,450) |  | Validation cohort  (N=880) |  | P- value |
| --- | --- | --- | --- | --- | --- | --- |
| **Opioid overdose** |  | 690 (20.0) |  | 176 (20.0) |  | 1 |
| **Age group, Mean ± SD** |  | 60.5 ± 17.8 |  | 60.1 ± 18.3 |  | 0.457 |
| 20~50 |  | 929 (26.9) |  | 226 (25.7) |  | 0.590 |
| 50~75 |  | 1,614 (46.8) |  | 409 (46.5) |  |  |
| ≥75 |  | 907 (26.3) |  | 245 (27.8) |  |  |
| **Male** |  | 1,601 (46.4) |  | 393 (44.7) |  | 0.353 |
| **CCI score, Mean ± SD** |  | 3.1 ± 2.7 |  | 3.0 ± 2.7 |  | 0.936 |
| 0~2 |  | 1,785 (51.7) |  | 456 (51.8) |  | 0.665 |
| 3~4 |  | 798 (23.1) |  | 205 (23.3) |  |  |
| 5~6 |  | 462 (13.4) |  | 117 (13.3) |  |  |
| ≥7 |  | 405 (11.7) |  | 102 (11.6) |  |  |
| **Health insurance type** |  |  |  |  |  |  |
| Medical insurance |  | 3,077 (89.2) |  | 774 (88) |  | 0.298 |
| Medical aid or NMS |  | 373 (10.8) |  | 106 (12) |  |  |
| **Cancer** |  |  |  |  |  |  |
| No |  | 2,719 (78.8) |  | 693 (78.8) |  | 0.999 |
| Non-metastatic cancer |  | 513 (14.9) |  | 131 (14.9) |  |  |
| Metastatic cancer |  | 218 (6.3) |  | 56 (6.4) |  |  |
| **Comorbid disease** |  |  |  |  |  |  |
| Substance use disorder |  | 44 (1.3) |  | 12 (1.4) |  | 0.836 |

SD, standard deviation; CCI, Charlson comorbidity index; NMS, National Meritorious Service

Supplementary table 4. Univariable logistic regression results for each variable

| **Variables, N (%)** | OR (95% CI) |  | P-value |
| --- | --- | --- | --- |
| **Age group** |  |  |  |
| 20~50 | Reference |  |  |
| 50~75 | 3.19 (2.41–4.22) |  | <0.001 |
| ≥75 | 7.00 (5.26–9.33) |  | <0.001 |
| **Male** | 1.04 (0.88–1.22) |  | 0.682 |
| **CCI score** |  |  |  |
| 0~2 | Reference |  |  |
| 3~4 | 3.12 (2.50–3.90) |  | <0.001 |
| 5~6 | 4.50 (3.52–5.76) |  | <0.001 |
| ≥7 | 5.14 (3.98–6.62) |  | <0.001 |
| **Health insurance type** |  |  |  |
| Medical insurance | Reference |  |  |
| Medical aid or NMS | 3.08 (2.45–3.86) |  | <0.001 |
| **Cancer** |  |  |  |
| No | Reference |  |  |
| Non-metastatic cancer | 1.59 (1.27–1.98) |  | <0.001 |
| Metastatic cancer | 1.97 (1.45–2.67) |  | <0.001 |
| **Comorbid disease** |  |  |  |
| Mood disorder | 3.02 (2.51–3.63) |  | <0.001 |
| Anxiety | 2.74 (2.29–3.28) |  | <0.001 |
| Schizophrenia | 4.28 (2.53–7.25) |  | <0.001 |
| Substance use disorder | 6.57 (3.56–12.13) |  | <0.001 |
| Myocardial infarction | 1.34 (0.77–2.34) |  | 0.299 |
| Heart failure | 2.47 (1.92–3.18) |  | <0.001 |
| Diabetes mellitus | 2.26 (1.91–2.68) |  | <0.001 |
| Hypertension | 3.02 (2.53–3.61) |  | <0.001 |
| Peripherral vascular disease | 1.98 (1.63–2.40) |  | <0.001 |
| Cerebrovascular disease | 4.84 (3.99–5.87) |  | <0.001 |
| Dementia | 3.79 (3.06–4.70) |  | <0.001 |
| Pancreatitis | 1.57 (1.14–2.16) |  | 0.006 |
| Respiratory disease | 1.36 (1.15–1.60) |  | <0.001 |
| Rheumatic disease | 1.42 (1.04–1.94) |  | 0.029 |
| Peptic ulcer disease | 1.45 (1.22–1.72) |  | <0.001 |
| Renal disease (severe) | 1.21 (0.69–2.13) |  | 0.504 |
| **No. of emergency department visit at baseline** | | | |
| 0 | Reference |  |  |
| 1~3 | 2.29 (1.91–2.76) |  | <0.001 |
| ≥4 | 5.77 (3.53–9.42) |  | <0.001 |
| **Cumulative duration of admission at baseline** | | | |
| 0 | Reference |  |  |
| 1~7 | 1.50 (1.18–1.90) |  | <0.001 |
| ≥8 | 2.92 (2.41–3.53) |  | <0.001 |
| **Concurrent medication** |  |  |  |
| Antianxiolytics | 2.25 (1.48–3.43) |  | <0.001 |
| Anticonvulsant | 6.66 (4.71–9.43) |  | <0.001 |
| Antidepressants | 3.03 (2.46–3.74) |  | <0.001 |
| Antipsychotics | 4.12 (3.1–5.49) |  | <0.001 |
| Benzodiazepines | 2.79 (2.3–3.38) |  | <0.001 |
| Gabapentinoids | 3.14 (2.49–3.96) |  | <0.001 |
| Muscle relaxant | 1.53 (1.25–1.86) |  | <0.001 |
| Non-opioid analgesics | 1.70 (1.42–2.05) |  | <0.001 |
| Other hypnotics | 2.83 (2.17–3.69) |  | <0.001 |
| Tramadol | 2.16 (1.8–2.59) |  | <0.001 |
| ***NIOA use pattern*** |  |  |  |
| **Cause of initiations** |  |  |  |
| Other | Reference |  |  |
| Traumatic injury | 1.08 (0.86–1.37) |  | 0.502 |
| Surgery | 0.70 (0.56–0.87) |  | 0.002 |
| **Ingredients** |  |  |  |
| Buprenorphine | 2.07 (1.64–2.61) |  | <0.001 |
| Codeine | 0.84 (0.67–1.05) |  | 0.123 |
| Dihydrocodeine | 1.28 (0.54–3.00) |  | 0.577 |
| Fentanyl | 2.28 (1.80–2.88) |  | <0.001 |
| Hydrocodone | 0.54 (0.16–1.82) |  | 0.323 |
| Hydromorphone | 1.48 (0.86–2.56) |  | 0.158 |
| Morphine | 3.13 (1.16–8.45) |  | 0.024 |
| Oxycodone | 2.83 (2.25–3.57) |  | <0.001 |
| Tapentadol | 1.34 (0.60–2.99) |  | 0.479 |
| **No. of ER/LA opioid** |  |  |  |
| 0 | Reference |  |  |
| 1 | 2.60 (2.16–3.12) |  | <0.001 |
| ≥2 | 3.99 (2.76–5.77) |  | <0.001 |
| **Persistence of use before index date** | | | |
| No | Reference |  |  |
| Past | 1.33 (0.98–1.8) |  | 0.069 |
| New | 1.77 (1.44–2.19) |  | <0.001 |
| Persistent | 3.25 (2.60–4.08) |  | <0.001 |
| **No. of prescriber** |  |  |  |
| 1 | Reference |  |  |
| 2 | 2.49 (1.94–3.19) |  | <0.001 |
| ≥3 | 5.03 (3.29–7.71) |  | <0.001 |
| **No. of prescription** |  |  |  |
| 1~2 | Reference |  |  |
| 3~6 | 2.14 (1.71–2.69) |  | <0.001 |
| 7~9 | 3.71 (2.45–5.61) |  | <0.001 |
| ≥10 | 8.27 (5.45–12.56) |  | <0.001 |
| **Daily MME** |  |  |  |
| 0~19 | Reference |  |  |
| 20~49 | 2.63 (2.05–3.37) |  | <0.001 |
| ≥50 | 2.98 (2.27–3.89) |  | <0.001 |

OR, odds ratio; CI, confidence interval; CCI, Charlson comorbidity index; NMS, National Meritorious Service; NIOA, non-injectable opioid analgesics; ER/LA, extended-release and long-acting; MME, morphine milligram equivalent Supplementary table 5. Development of predictive risk score model (simple model)

| Risk factor |  | Univariable OR (95% CI) |  | Multivariable aOR (95% CI) | β coefficient | Score |
| --- | --- | --- | --- | --- | --- | --- |
| **Age group** |  |  |  |  |  |  |
| 20~50 |  | Reference |  | Reference | - | 0 |
| 50~75 |  | 3.19 (2.41–4.22) |  | 2.11 (1.56–2.86) | 0.75 | 7 |
| ≥75 |  | 7.00 (5.26–9.33) |  | 3.38 (2.44–4.68) | 1.22 | 12 |
| **Health insurance type** |  |  |  |  |  |  |
| Medical insurance |  | Reference |  | Reference | - | 0 |
| Medical aid or NMS |  | 3.08 (2.45–3.86) |  | 1.96 (1.52–2.54) | 0.67 | 7 |
| **Comorbidities** |  |  |  |  |  |  |
| CVD |  | 4.84 (3.99–5.87) |  | 2.57 (2.05–3.22) | 0.94 | 9 |
| Dementia |  | 3.79 (3.06–4.70) |  | 1.31 (1.01–1.7) | 0.27 | 3 |
| **Concurrent medication** |  |  |  |  |  |  |
| Anticonvulsants |  | 6.66 (4.71–9.43) |  | 4.83 (3.25–7.19) | 1.58 | 16 |
| Benzodiazepine |  | 2.79 (2.30–3.38) |  | 1.63 (1.31–2.04) | 0.49 | 5 |
| Gabapentinoids |  | 3.14 (2.49–3.96) |  | 1.89 (1.45–2.46) | 0.63 | 6 |
| Tramadol |  | 2.16 (1.80–2.59) |  | 1.26 (1.02–1.55) | 0.23 | 2 |
| **Cause of analgesics** |  |  |  |  |  |  |
| Other ^a^ |  | Reference |  | Reference | - | 0 |
| Surgery |  | 0.69 (0.56–0.86) |  | 0.57 (0.45–0.73) | -0.55 | -6 |
| **No. of ER/LA opioid** |  |  |  |  |  |  |
| 0 |  | Reference |  | Reference | - | 0 |
| ≥1 |  | 2.76 (2.32–3.28) |  | 1.98 (1.62–2.41) | 0.68 | 7 |

OR, odds ratio; aOR, adjusted odds ratio; CI, confidence interval; NMS, National Meritorious Service; CVD, cerebrovascular disease; ER/LA, extended-release and long-acting
^a^ Traumatic injury was collapsed into other category
^b^ For Multivariable logistic regression model, ≥2 ER/LA opioid was collapsed into ≥1 ER/LA opioid

Supplementary table 6. Prediction performance stratified by risk (simple model)

| Performance metrics (Simple model) | Development cohort | | |  | Validation cohort | | |
| --- | --- | --- | --- | --- | --- | --- | --- |
|  | Low risk | Intermediate risk | High risk |  | Low risk | Intermediate risk | High risk |
| Total, N (%) | 1,957 (56.7) | 1,128 (32.7) | 365 (10.6) |  | 490 (55.7) | 303 (34.4) | 87 (9.9) |
| Predicted score (range) | (-6~12) | (13~27) | (28~75) |  | (-6~12) | (13~27) | (28~75) |
| Actual overdose episodes, N (% of each subgroup) | 136 (6.9) | 350 (31.0) | 204 (55.9) |  | 36 (7.3) | 84 (27.7) | 56 (64.4) |
| Actual non-overdose episodes, N (% of each subgroup) | 1,821 (93.1) | 778 (69.0) | 161 (44.1) |  | 454 (92.7) | 219 (72.3) | 31 (35.6) |
| Actual severe overdose episodes, N (% of each subgroup) | 34 (1.7) | 112 (9.9) | 77 (21.1) |  | 12 (2.4) | 23 (7.6) | 22 (25.3) |
| Actual non-severe overdose episodes, N (% of each subgroup) | 1,923 (98.3) | 1,016 (90.1) | 288 (78.9) |  | 478 (97.6) | 280 (92.4) | 65 (74.7) |
| Sensitivity, % (95% CI) | - | 80.3 (77.1–83.2) | 29.6 (26.2–33.1) |  | - | 79.6 (72.8–85.2) | 31.8 (25.0–39.3) |
| Specificity, % (95% CI) | - | 66.0 (64.2–67.8) | 94.2 (93.2–95.0) |  | - | 64.5 (60.8–68.0) | 95.6 (93.8–97.0) |
| LR (+) (95% CI) | - | 2.36 (2.21–2.52) | 5.07 (4.20–6.12) |  | - | 2.24 (1.98–2.54) | 7.23 (4.81–10.85) |
| LR (-) (95% CI) | - | 0.30 (0.26–0.35) | 0.75 (0.71–0.79) |  | - | 0.32 (0.24–0.43) | 0.71 (0.64–0.79) |
| OR (95% CI) | Reference | 6.02 (4.86–7.47) | 17.00 (12.95–22.23) |  | Reference | 4.84 (3.17–7.38) | 22.78 (13.08–39.67) |
| % of all overdose episodes captured ^a^ | 19.7% | 50.7% | 29.6% |  | 20.5% | 47.7% | 31.8% |
| % of all severe overdose episodes captured ^b^ | 15.2% | 50.2% | 34.5% |  | 21.1% | 40.4% | 38.6% |

CI, confidence interval; LR, likelihood ratio; OR, odds ratio
^a^ development cohort: n=690; validation cohort: n=176
^b^ development cohort: n=223; validation cohort: n=57

Supplementary table 7. Prediction performance with strict outcome definition stratified by risk

| Performance metrics (Full model) |  | Full cohort | | |
| --- | --- | --- | --- | --- |
|  |  | Low risk | Intermediate risk | High risk |
| Total, N (%) |  | 1,785 (64.0) | 688 (24.7) | 317 (11.4) |
| Predicted score (range) |  | (-5~17) | (18~31) | (32~129) |
| Actual overdose episodes, N (% of each subgroup) |  | 132 (7.4) | 231 (33.6) | 195 (61.5) |
| Actual non-overdoseepisodes, N (% of each subgroup) |  | 1,653 (92.6) | 457 (66.4) | 122 (38.5) |
| Actual severe overdose episodes, N (% of each subgroup) |  | 36 (2.0) | 70 (10.2) | 69 (21.8) |
| Actual non-severe overdose episodes, N (% of each subgroup) |  | 1,749 (98.0) | 618 (89.8) | 248 (78.2) |
| Sensitivity, % (95% CI) |  | - | 76.3 (72.6–79.8) | 34.9 (31.0–39.1) |
| Specificity, % (95% CI) |  | - | 74.1 (72.2–75.9) | 94.5 (93.5 – 95.4) |
| LR (+) (95% CI) |  | - | 2.94 (2.71–3.20) | 6.39 (5.20–7.86) |
| LR (-) (95% CI) |  | - | 0.32 (0.27–0.37) | 0.69 (0.65–0.73) |
| OR (95% CI) |  | Reference | 6.33 (4.99–8.03) | 20.02 (15.02–26.68) |
| % of all overdose episodes captured (n=558) |  | 23.7% | 41.4% | 34.9% |
| % of all severe overdose episodes captured (n=175) |  | 20.6% | 40.0% | 39.4% |

CI, confidence interval; LR, likelihood ratio; OR, odds ratio

Supplementary table 8. Prediction performance with strict outcome definition stratified by risk (simple model)

| Performance metrics (Simple model) |  | Full cohort | | |
| --- | --- | --- | --- | --- |
|  |  | Low risk | Intermediate risk | High risk |
| Total, N (%) |  | 1.484 (53.2) | 982 (35.2) | 324 (11.6) |
| Predicted score (range) |  | (-6~12) | (13~27) | (28~75) |
| Actual overdose episodes, N (% of each subgroup) |  | 96 (6.5) | 275 (28.0) | 187 (57.7) |
| Actual non-overdose episodes, N (% of each subgroup) |  | 1,388 (93.5) | 707 (72.0) | 137 (42.3) |
| Actual severe overdose episodes, N (% of each subgroup) |  | 27 (1.8) | 77 (7.8) | 71 (21.9) |
| Actual non-severe overdose episodes, N (% of each subgroup) |  | 1,457 (98.2) | 905 (92.2) | 253 (78.1) |
| Sensitivity, % (95% CI) |  | - | 82.8 (79.4–85.8) | 33.5 (29.6–37.6) |
| Specificity, % (95% CI) |  | - | 62.2 (60.1–64.2) | 93.9 (92.8–94.8) |
| LR (+) (95% CI) |  | - | 2.19 (2.05–2.34) | 5.46 (4.47–6.67) |
| LR (-) (95% CI) |  | - | 0.28 (0.23–0.33) | 0.71 (0.67–0.75) |
| OR (95% CI) |  | Reference | 5.62 (4.38–7.22) | 19.74 (14.59–26.70) |
| % of all overdose episodes captured (n=558) |  | 17.2% | 49.3% | 33.5% |
| % of all severe overdose episodes captured (n=175) |  | 15.4% | 44.0% | 40.6% |

CI, confidence interval; LR, likelihood ratio; OR, odds ratio


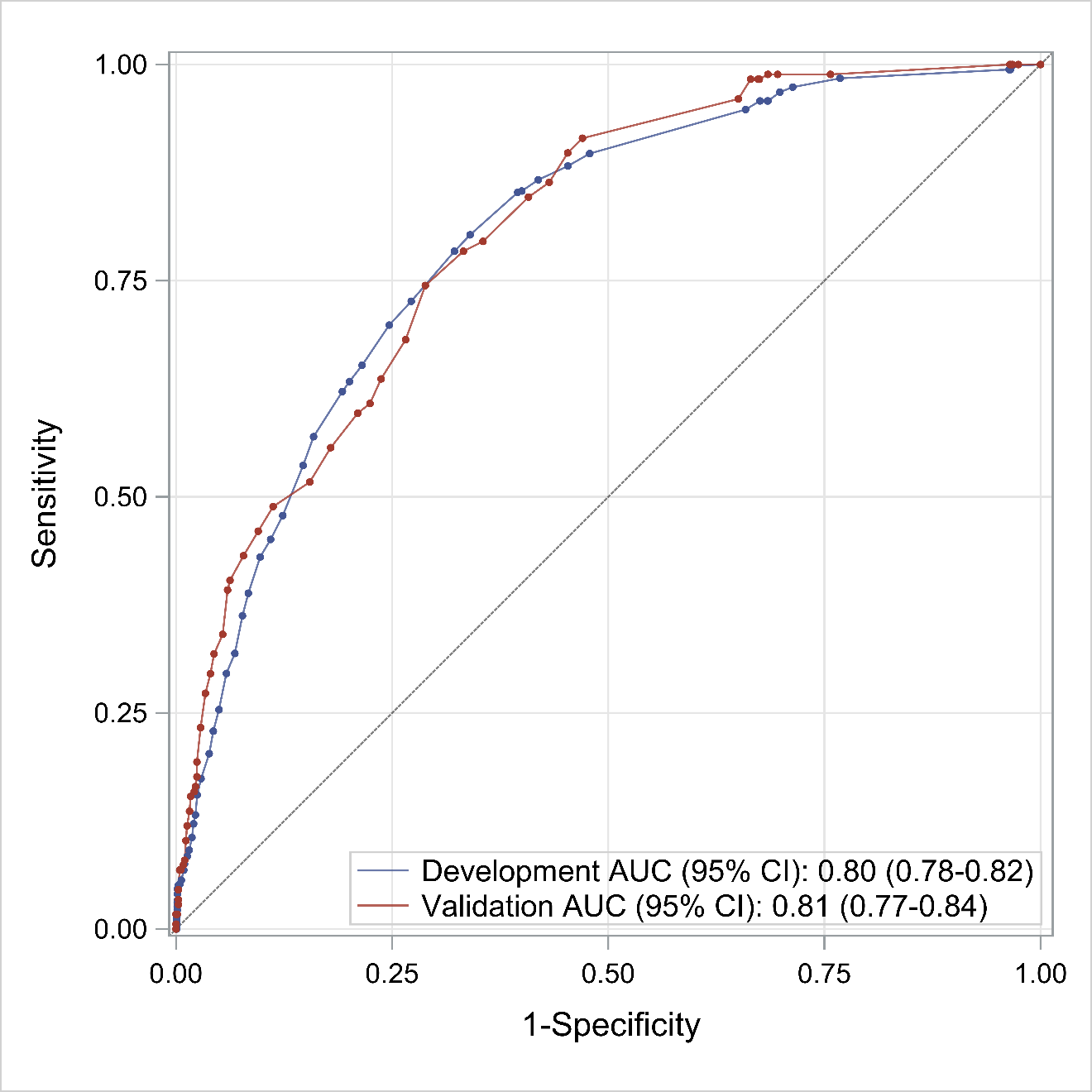


Supplementary figure 1. Receiver operating characteristic curve of predictive risk score model (simple model) for opioid overdose

AUC, area under the receiver operating characteristic curve; CI, confidence interval


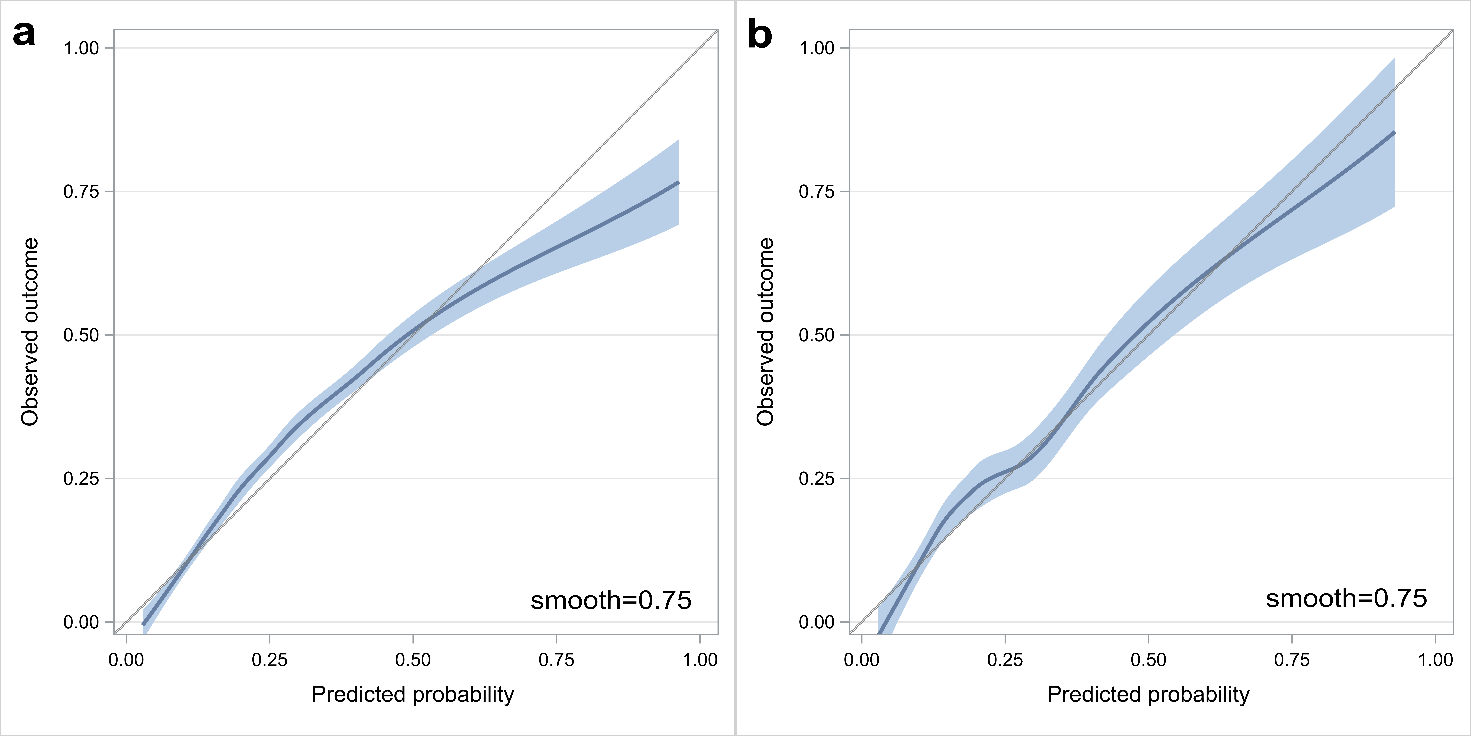


Supplementary figure 2. Calibration plot for predictive risk score model (simple model) for opioid overdose

1. Calibration plot in the development cohort. (b) Calibration plot in the validation cohort.


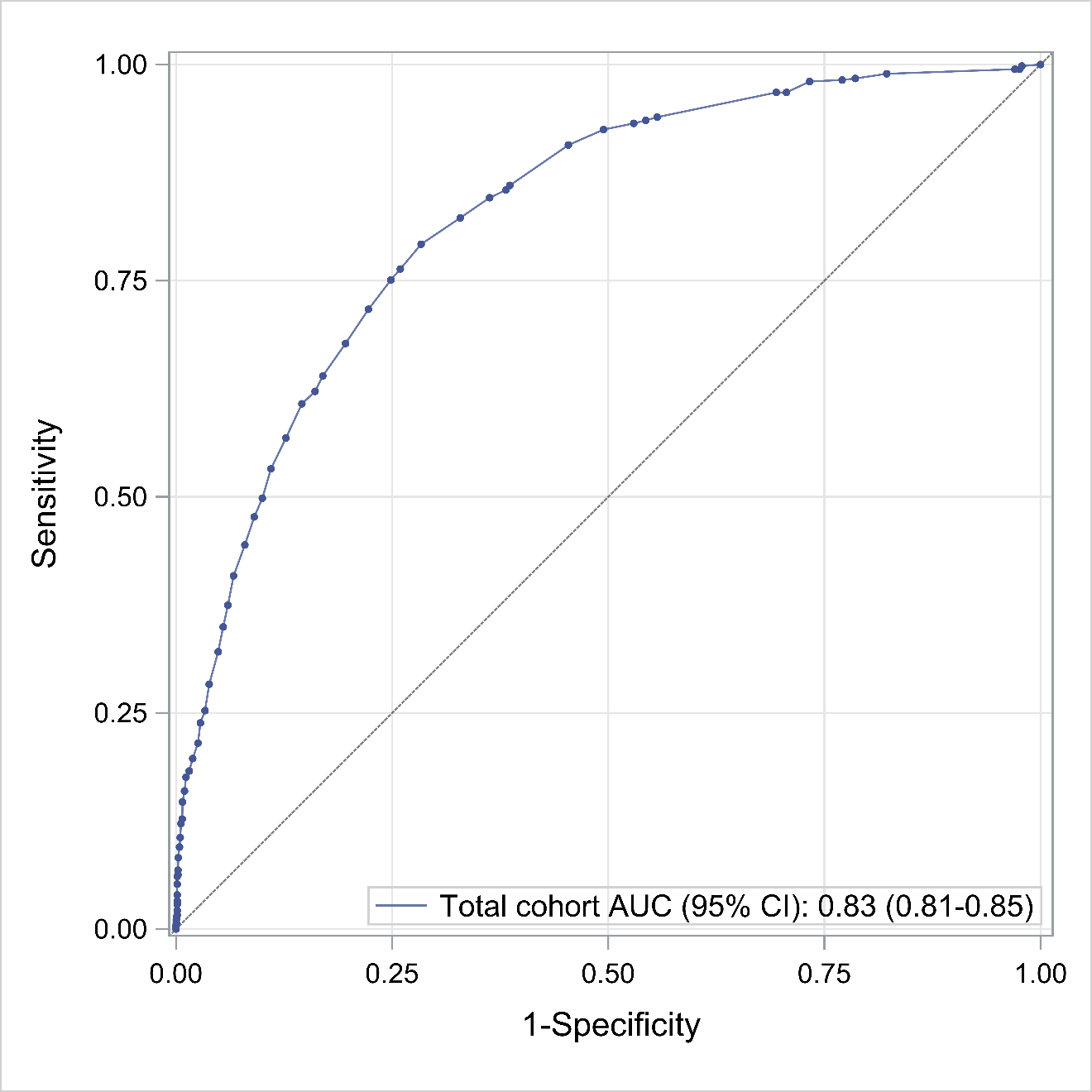


Supplementary figure 3. Receiver operating characteristic curve of predictive risk score model for opioid overdose with strict outcome definition

AUC, area under the receiver operating characteristic curve; CI, confidence interval


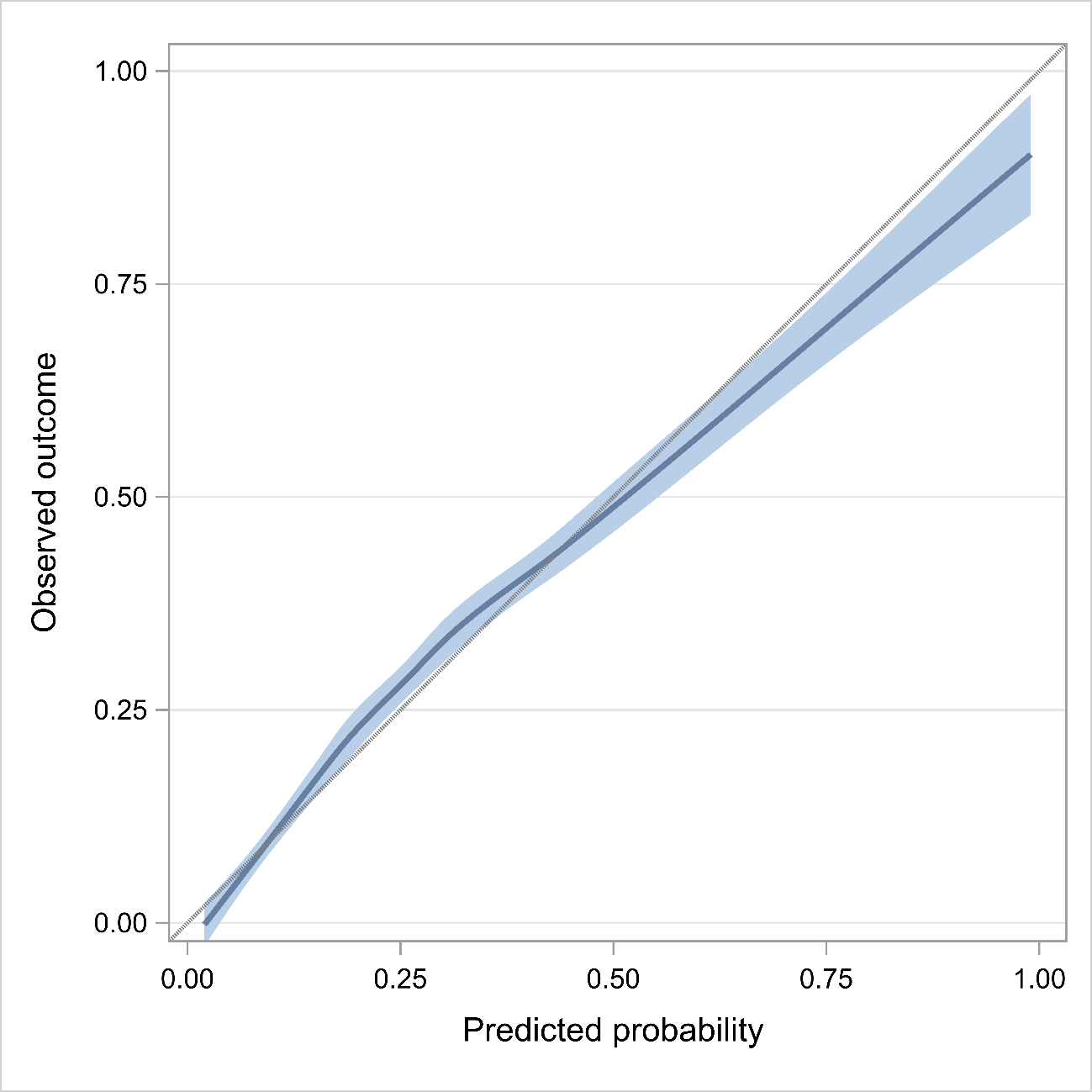


Supplementary figure 4. Calibration plot for predictive risk score model for opioid overdose with strict outcome definition


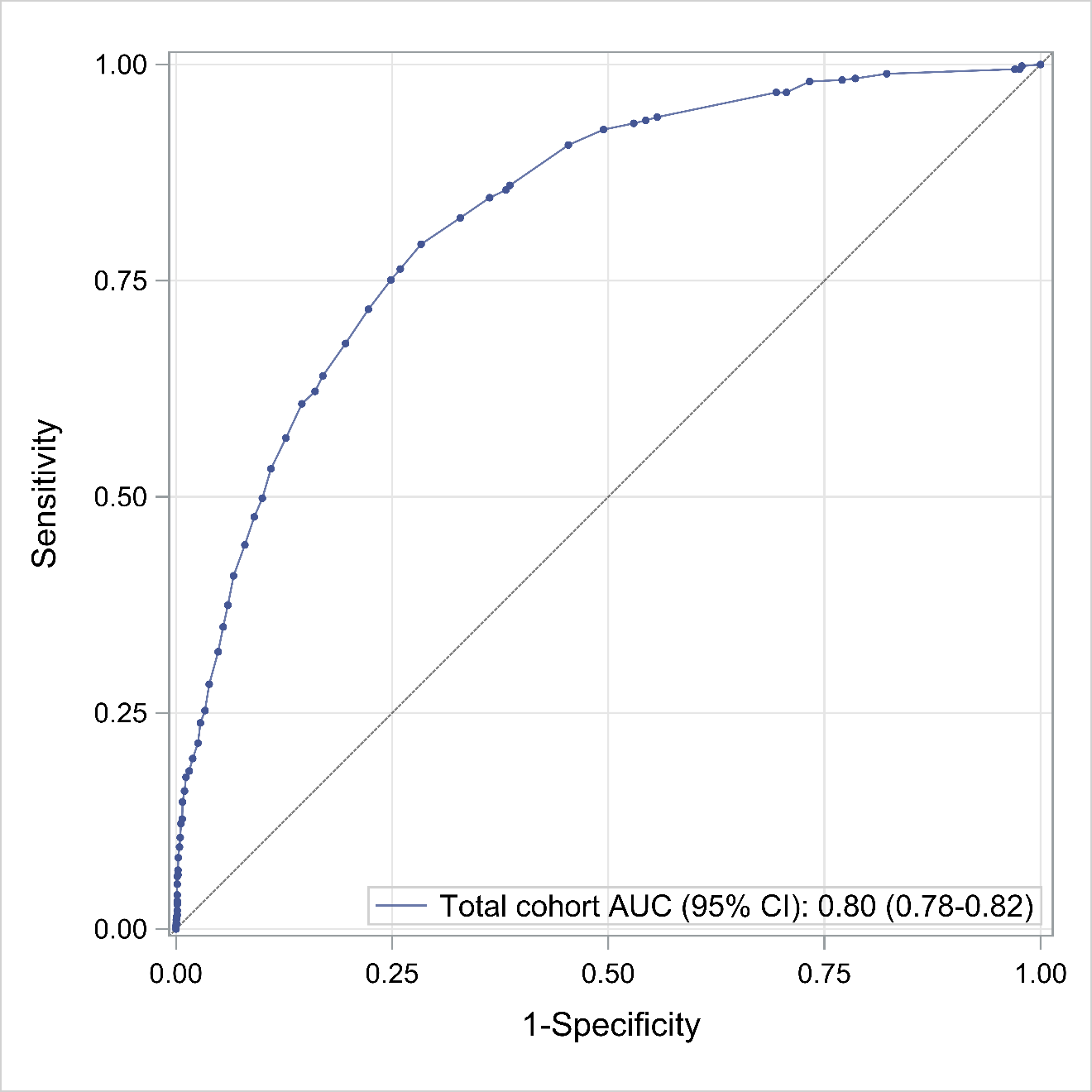


Supplementary figure 5. Receiver operating characteristic curve of predictive risk score model (simple model) for opioid overdose with strict outcome definition

AUC, area under the receiver operating characteristic curve; CI, confidence interval


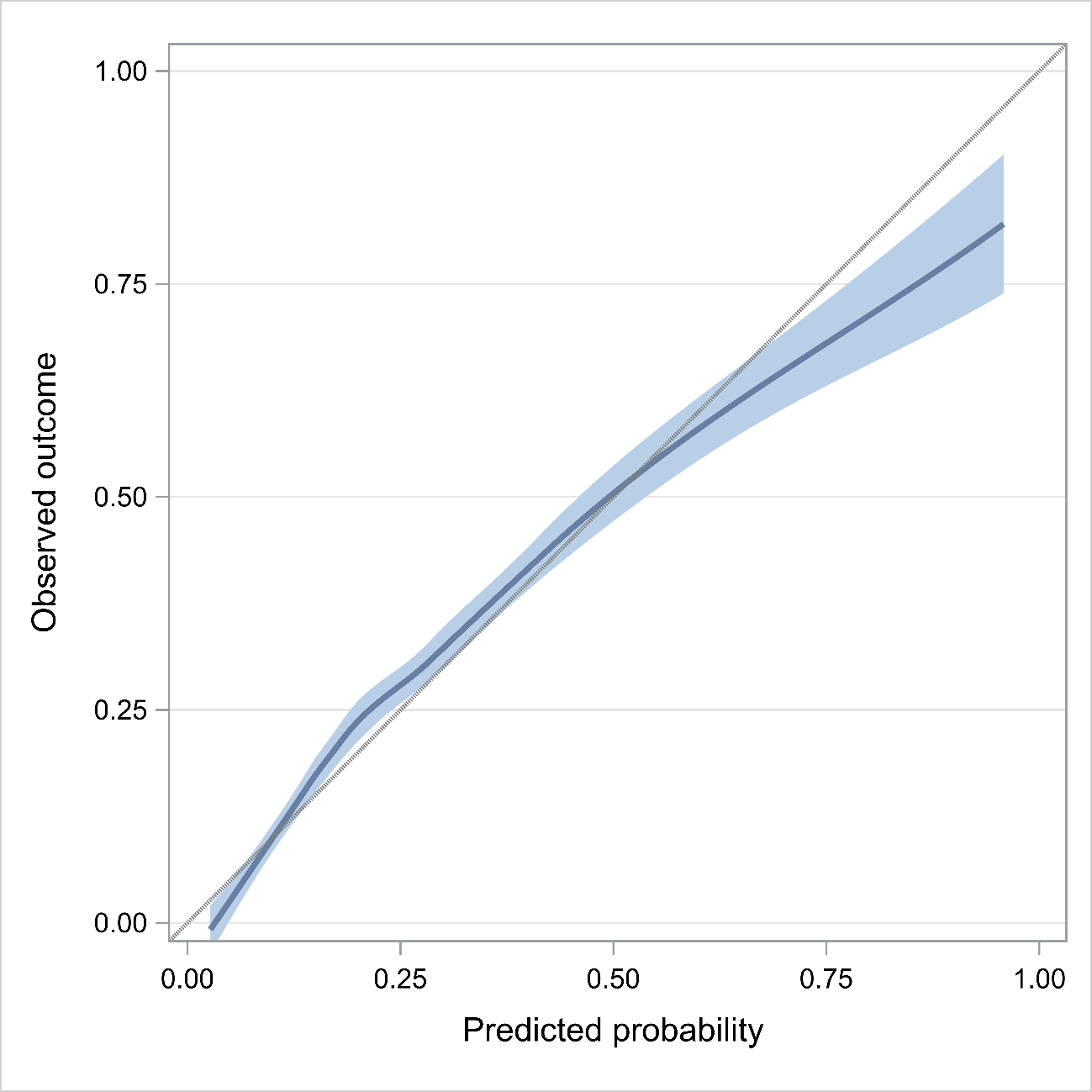


Supplementary figure 6. Calibration plot for predictive risk score model (simple model) for opioid overdose with strict outcome definition
